# Dengue and Relative Bradycardia

**DOI:** 10.3201/eid1402.070401

**Published:** 2008-02

**Authors:** Sanjaya Naresh Senanayake

**Affiliations:** * The Canberra Hospital – Infectious Diseases, Woden, Australian Capital Territory, Australia

**Keywords:** Dengue, bradycardia, letter

**To the Editor:** In a recent letter to Emerging Infectious Diseases, Lateef and colleagues identified a relationship between dengue and relative bradycardia in patients in Singapore. They stated that “To our knowledge, this sign has not been previously associated with dengue” (*1*). Unfortunately, the association of dengue fever with relative bradycardia has already been well established and is certainly not a new finding (*2*,*3*). Despite this, however, there is no harm done in reinforcing an often forgotten clinical sign that can assist in the diagnosis of dengue, especially in those countries with limited resources.
